# Self-encapsulation liquid metal materials for flexible and stretchable electrical conductors†

**DOI:** 10.1039/c9ra06098g

**Published:** 2019-10-30

**Authors:** Jun-Heng Fu, Jian-Ye Gao, Sen Chen, Peng Qin, Jin-Tao Shi, Jing Liu

**Affiliations:** CAS Key Laboratory of Cryogenics, Technical Institute of Physics and Chemistry Beijing 100190 China jliu@mail.ipc.ac.cn; School of Future Technology, University of Chinese Academy of Sciences Beijing 100049 China; Beijing Key Laboratory of Cryo-Biomedical Engineering Beijing 100190 China; Department of Biomedical Engineering, School of Medicine, Tsinghua University Beijing 100084 China

## Abstract

A one-step strategy for fabricating flexible conductors *via* phase separation is proposed, wherein, the liquid metal was implanted into polydimethylsiloxane, whose viscosity was changed using hexane. Such self-encapsulating composite exhibited good electronic and mechanical stability under mechanical cycles with no significant leaking of droplets during the testing process.

## Abstract 2

A one-step strategy for fabricating flexible conductors *via* phase separation is proposed, wherein, the liquid metal was implanted into polydimethylsiloxane, whose viscosity was changed using hexane.

## Graphical abstract

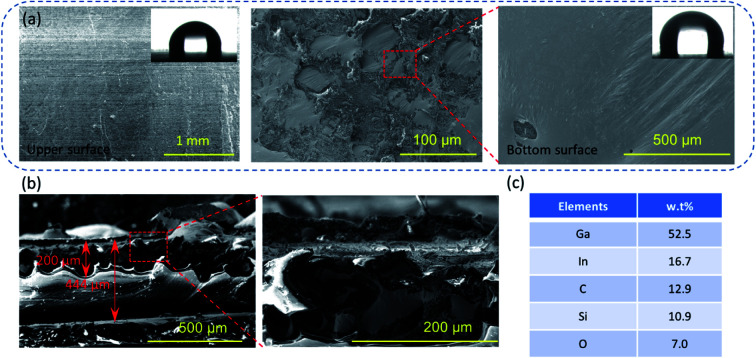

Soft composites with excellent stretchability and electrical conductivity have a strong presence in the fields of electronic skin,^1–4^ flexible displays,^5–7^ and soft robotics.^8,9^ In these applications, it is a general approach to fabricate a flexible conductive composite by encapsulating electrical materials in a permeable polymer matrix, such as noble metal micro/nano particles,^10–12^ carbon black,^13,14^ and graphene/carbon nanotube materials.^15–17^ These embedded materials, which are generally rigid and brittle, have difficulty in matching with each other and mismatch with the matrix because of stress concentration. Furthermore, with the development of additive manufacturing, different types of printing methods have emerged, which led to the result that the quest for insulative packaging is increasing.^18–22^ Overall, the properties of the as-prepared composites are not only affected by the uniform distribution of the conductive material in the polymer matrix, but also with the choice of the packaging material. Therefore, the demand for a material tightly integrated to the polymer substrate as a conductive phase provides potential for the development of high-stability flexible electronics and devices.

In order to obtain a flexible self-encapsulating composite, the choice of an elastomer with good biosecurity and stability are fundamentally essential. Polydimethylsiloxane (PDMS) is widely used due to its chemical stability and low-cost preparation process, as well as flexibility and transparency.^16^ A flexible package of PDMS films with different surface microstructures has been used in stress sensing and^15,16^ microfluidics,^23,24^ which has been prepared by means of moulding, spin coating, and injection. These processes, however, can cause damages to the film and are often expensive. At the same time, to fabricate these films, we need to carefully prepare the PDMS film to be peeled off from the substrate, which demands for high requirements on the preparation process.

Room temperature liquid metals with good rheological properties, metal conduction and thermal conductivity are increasingly used in thermal management,^25,26^ energy storage,^27–29^ composite materials,^30–32^ and electronic printing.^18,20,33^ Using liquid metals as electrical components filled in an elastomer to constitute a conductive elastomer is receiving widespread attention. For example, liquid metals are filled in a carboxylated polyurethane (CPU) to prepare self-healing energy storage devices.^34^ Chen *et al.* proposed to mix a liquid metal with silicone oil to obtain a smart material that can achieve conductor–insulator transition.^35^ In addition, liquid metal elastomers have the functions of the wave-absorbing and dielectricity as well.^36,37^ However, the high cost of liquid metals limits their extensive usage. Moreover, droplet leakage due to the mobility of liquid metal still remains a technical barrier in these applications. Therefore, it is indispensable to reduce the dosage of liquid metals and their ability to leak in the composite.

There are generally two approaches to obtain flexible elastomers by embedding liquid metals. The first way is to eliminate the isolation phenomenon of the liquid metal elastomer by adding a highly conductive material such as carbon nanotubes,^38^ graphene,^39^ graphite and precious metal materials.^40^ The isotropic homogeneity of multiphase conductive materials in elastic polymers and the electronical and mechanical stability are still challenges during cycling tests. Besides, the leakage of liquid metal droplets also remains unsolved. Another mechanism of connecting liquid metal droplets in polymers is by applying an external force, such as stretching,^25,41^ scratching^42^ and freezing,^35,43^ to form a conductive path. The advantage of this method is that these materials can be directly applied to sensors by measuring the variation of pressure, temperature and humidity, and this strategy settles the leakage by wrapping the droplets with a polymer. Such method, however, requires specific working conditions, which constrains its versatility. In this study, combining the advantages of the two strategies, a conductive elastomer only composed of PDMS and liquid metal with good electrical and mechanical properties was fabricated meanwhile overcoming the shortcomings of the existing strategies. The Janus structure, which consists of a conducive liquid metal phase and encapsulating polymer phase, works normally under various mechanical loading.

Herein, we propose a one-step method to develop a self-encapsulation conductor based on the gravity deposition of liquid metals and PDMS. Because of the different densities of the two materials, phase separation of the mixed composites will occur, wherein the light PDMS phase is placed above the LM phase, which naturally encapsulates the liquid metal. The resistance of the resultant composite is almost 10 mΩ sq^−1^, and the measure of electronic and mechanical properties illustrated the favourable flexibility and stable electronics by recording the relative resistance changes when applying repeated twisting and bending cycles. This composite based on PDMS/hexane@liquid metal can introduce a brand-new perspective and contribute to the change and optimization in the fabrication and encapsulation of conductors.

The preparation of a liquid metal self-packaging conductor is shown in Scheme 1. The liquid metal mentioned here is composed of 75.5% gallium and 24.5% indium by weight, prepared by heating (150 °C) and mixing *via* a mechanical stirrer for 1.5 hour. The PDMS and hexane are used as packaging materials, where the presence of hexane helps reduce the viscosity of PDMS so that phase separation can occur more easily when mixed with the liquid metal, avoiding the formation of islands of liquid metal particles. The specific preparation method is as follows. PDMS (base : curing agent = 10 : 1) was degassed and mixed with hexane, and vigorously stirred. The resultant mixture was mixed with the liquid metal using a mechanical stirrer for 5 min at a speed of 600 rmp. Then, the resulting material was transferred into a vacuum chamber to remove bubbles, and kept at room temperature for 30 minutes. The cured PDMS-liquid metal conductor was fabricated at the temperature of 70 °C in 4 hours. After solidification, a self-encapsulating liquid metal composite (SELMC) with a certain thickness was obtained, and the thickness of the electrical conductor can be regulated by adjusting the content of the conductive solution. In this study, hexane was chosen to help solve the problem of the formation of liquid metal islands. By changing the viscosity of the composite,^44,45^ the liquid metal droplets naturally settle to the bottom under the action of gravity. Besides, hexane volatilization further reduces the content of the bottom polymer due to its low boiling point (68 °C), causing the composite to exhibit delamination during the curing process. The elastic modulus of the SELMC obtained by mixing different volumes of liquid metal with PDMS is as shown in Fig. S1 (ESI†), where its Young's modulus is between 0.95 and 2.13 MPa. When the volume ratio of PDMS/LM is 4 : 1, the strain can reach 95%, showing good tensile properties. When the volume ratio is 9 : 1, it shows good hardness, which can withstand 1.6 MPa pressure, although the strain is 75%. Here, considering the deformation and stress tolerance, the PDMS-hexane solution and liquid metal with the volume ratio of 4 : 1 are chosen. The prepared stretchable electrical conductor can be tailored to obtain various shapes, as shown in Scheme 1b–e.

**Scheme 1:**
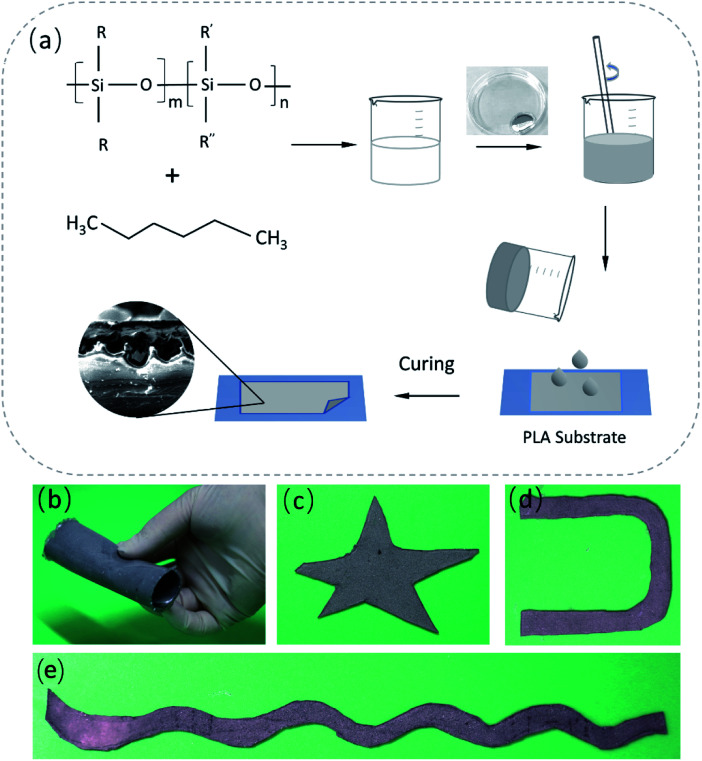
(a) Scheme illustration of the fabrication process of the flexible and stretchable SELMC, and photographs of the different shapes of the SELMC (b) flexible and rolled sheet, (c) star pattern, (d) U shape, and (e) strip pattern.

The microscopy images of environmental scanning electron microscopy (ESEM; QUANTA FEG 250, America) demonstrated that the upper and bottom surfaces of the flexible conductor have different microstructures, as shown in Fig. 1a and b. The smooth upper surface is a polymer layer with an aligned layered structure. At the same time, there are some pore-like structures (see in Fig. S2, ESI†). This is because hexane evaporates and leaves a certain pore structure when the curing temperature is above 68 °C. For the liquid metal layer, as the conductive phase, the exposed liquid metal is oxidized to Ga_2_O_3_, which has an irregular morphology of convex curls, but the entire oxide layer is integrally connected, and avoids the formation of an island of liquid metal droplets. Fig. S3 (ESI†) displays the microstructure of the liquid metal embedded elastomer without hexane doping, which presented the serious oxidation of liquid metal (silvery area) and discreteness between liquid metal droplets due to the oxidation. This result indicates that hexane contributes to the phase separation and forms stable self-encapsulation materials.

**Fig. 1:**
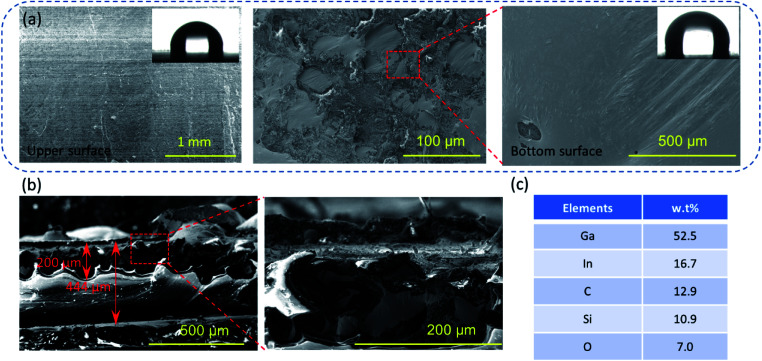
(a and b) the SEM images of the flexible and stretchable SELMC, and the inset in (a) is the contact angle, (c) the elemental distribution of SELMC in the section by EDS.

The contact angle (contact angle measurement instrument JC200D3) of the upper and bottom surfaces shows that the doping of the liquid metal in PDMS exhibits no significant changes in the roughness of the surface structure (the contact angle is 90° ± 1°), which evidences the surface flatness because of the liquid metal oxide film at the bottom. The entire conductor has a thickness of 444 μm, wherein the liquid metal layer has a thickness of 200 μm. The electrical resistance of the conductor is 10 mΩ sq^−1^. From the energy dispersive spectrometry (EDS; 6853-H, HORIBA, Ltd.) results, the metal and metal oxide phases account for 76.1 wt% in the liquid metal layer, which illustrates that the lesser amount of polymer serves only as a packaging layer. These results also show that the liquid metal is deposited at the bottom and is not isolated by the polymer into islands.

In order to test the electrical and mechanical properties of the SELMC, the electrical and mechanical performances were measured using a mechanical testing instrument. As shown in Fig. 2, the elastic parameters of the as-prepared sample are Young's modulus of 3.95 kPa and fracture strain of 57%. Compared with pure PDMS, the elastic modulus of the as-prepared SELMC is reduced by three orders of magnitude. We also prepared elastomers with different volume ratios, but only measured the stress–strain curve of the conductive material with a volume ratio of 4 : 1 since the one with a volume ratio of 12 : 1 is in a fluidic state. The elastic modulus results show that the hardness of the material increased due to the hardness of the composite system strengthened by an increased amount of liquid metal.^46^Fig. 3a shows the relative resistance change curve of the sample with a width of 13 mm during bending cycle measurements (90°). During the first bending cycle, the relative resistance of the sample after bending was 0.995. When it returns to the initial state, the relative resistance returns to the original state. In the following cycling tests, the resistance changes steadily, and there is only a slight difference between the bending and releasing process. We repeated the cycle 50 times and found that after 14 times of bending, the bending and releasing process had no effect on the resistance. Compared with the initial state, the maximum variation of the relative resistance was 10%. After 50 measurements, the stable resistance was 3.5 Ω. In order to obtain the change in the relative resistance of the flexible conductor when both stretching and bending are applied, the relative resistance patterns of samples in both bending and stretching states were tested by finger movements. As shown in Fig. 3b, the test sample (sample T1) was placed on a finger and fixed at both ends to obtain the strain along the longitudinal direction when the finger bends. During the 15 cycles, the releasing resistance of sample T1 did not change. The relative resistance slightly increased when the finger bended. The results show that the fabricated flexible SELMC has excellent mechanical stability. Moreover, there is no obvious leakage of liquid metal droplets during the whole stress testing process. The results can also be verified by subsequent electrical tests. Similar results can be obtained by selecting other samples T2 and T3 for the measurement as shown in Fig. S4 and S5 (ESI†). Furthermore, Fig. S6† shows the resistance of sample T2 over 2000 bending cycles, where it showed a stable performance.

**Fig. 2:**
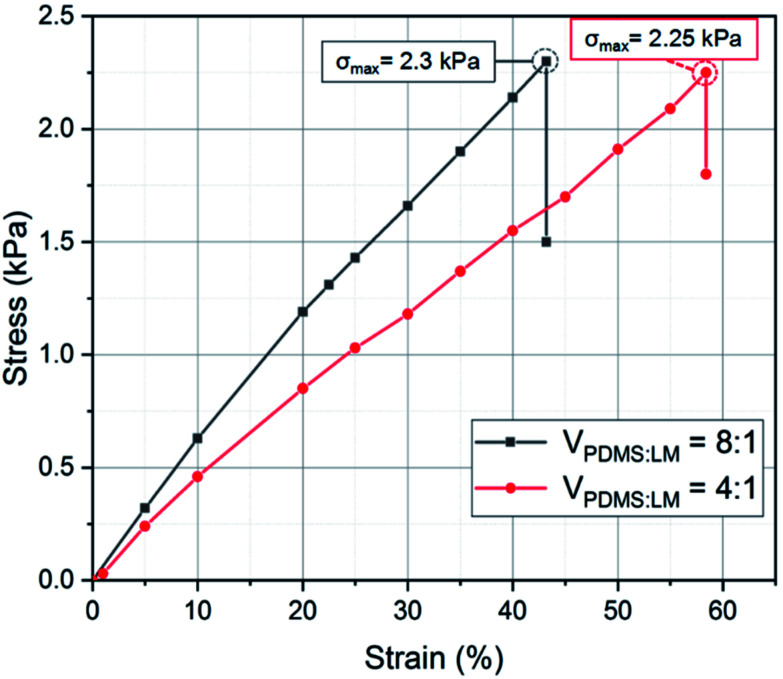
Tensile curve of the as-fabricated sample with different volume ratios.

**Fig. 3:**
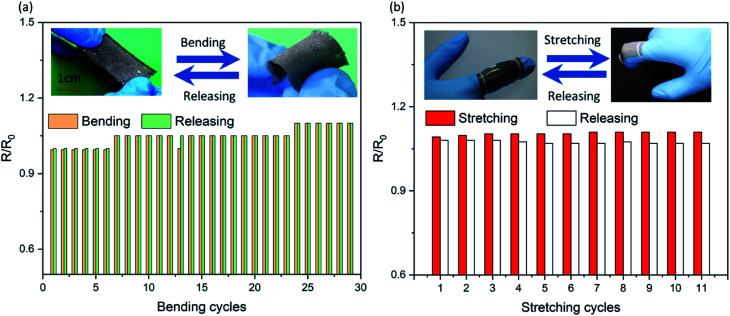
The relative resistance change curves of SELMC under (a) bending cycles and (b) the cooperation of bending and stretching cycles.

The temperature effect on the material caused by Joule heating of the circuit has a significant influence on the stability of the circuit. Fig. 4a describes the thermogravimetric analysis result of the elastic polymer, which shows a slight change in the presence of low boiling hexane compared to the pure PDMS. When the operating temperature is below 150 °C, both can be stably operated without thermal decomposition. Regardless of the presence of hexane, the trend of thermal degradation is synchronous as the temperature increases to 1000 °C. In addition, we placed a 5 cm × 5 cm square SELMC on a heating platform and measured the temperature difference between the upper and lower surfaces as the temperature increased. As shown in Fig. 4b, the upper and lower surfaces exhibited temperature anisotropy, and the maximum temperature difference was 40 °C (when the heating platform was 160 °C). The temperature anisotropy exhibited in the normal direction confirms that the liquid metal phase is at the bottom of the material and the upper surface is the polymer layer with poor thermal conductivity.

**Fig. 4:**
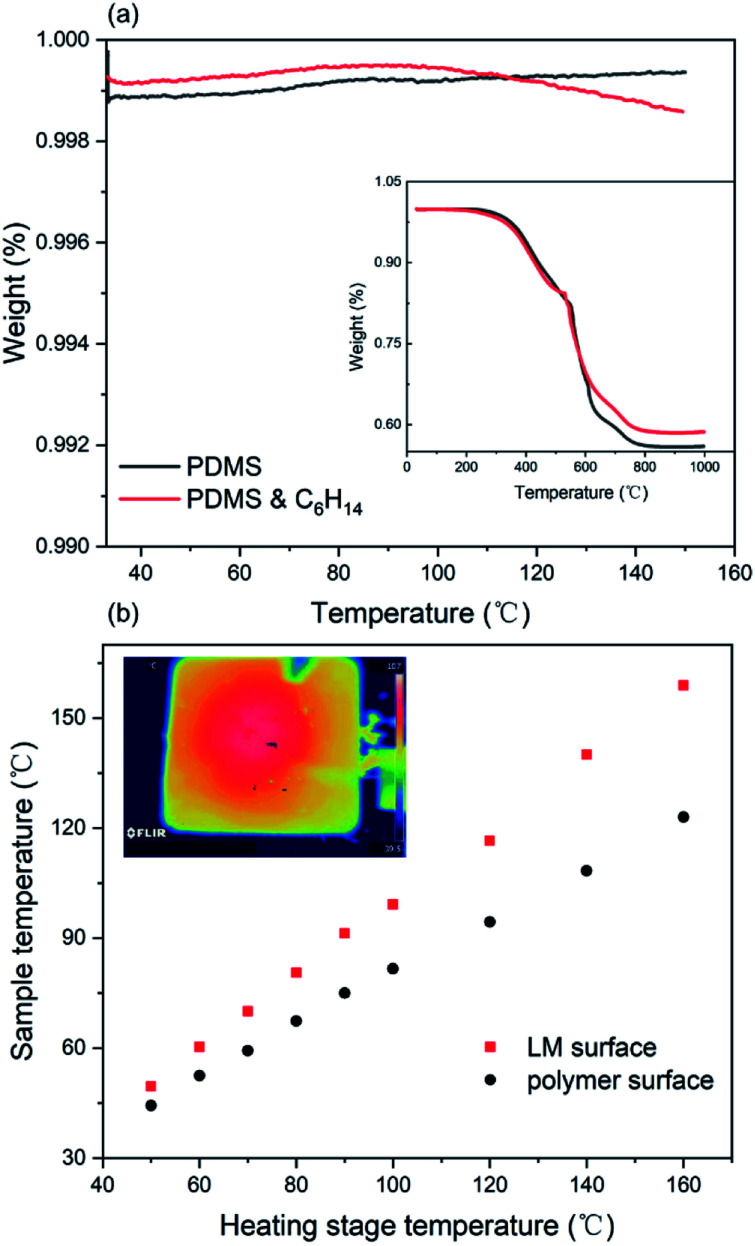
(a) The TGA curve of the PDMS and the mixture of the polymer and hexane, and (b) plot of the temperatures of the SELMC film *versus* the heating stage temperature.

In order to show the self-encapsulation characteristics of the sample intuitively, the sample was used as a conductor and connected to a LED lamp to display a circuit. As shown in Fig. 5a, when the power is connected to the upper surface of the sample (containing polymer), the circuit is disconnected, whereas, when it is connected to the lower surface (containing the conductive phase), the circuit works. The results show that the self-encapsulation circuit formed by the material under gravity is feasible. Fig. 5b and c show the working state of the circuit under different external forces, such as bending, stretching and twisting, and their cooperative effects. Under the condition of a continuous action of an external force, the circuit still works (see in Fig. S7, ESI†). It can be seen that no obvious liquid metal droplets penetrate even after multiple distortions. These results indicate that the self-encapsulating materials have stable mechanical, electrical and packaging properties.

**Fig. 5:**
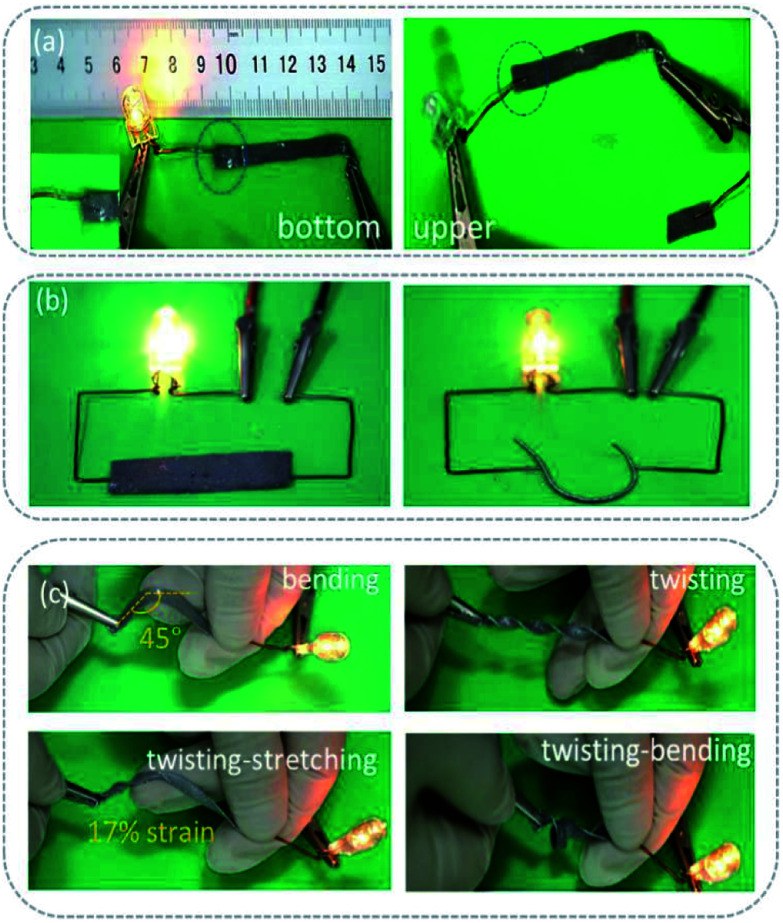
The photographs of electronical circuits (a) in the upper and lower surface of SELMC, (b) in strip and filamentous shapes of conductor, image (c) is the flexible electronic circuits under various deformation conditions in 45° bending state, twisting state, 17% strain twisting–stretching state, and twisting–bending state, respectively.

It is noteworthy that the excellent electrical conductivity exhibited by SELMC during the stretching process is unique to liquid metals. Up to now, conventional conductive composites are mostly composed of conductive rigid fillers and insulating substrates, which reduce the electrical conductivity of the material due to the conductive phase being isolated during the stretching process. Liquid metals, as a conductive phase that can flow and deform, have been widely used in stretchable conductive materials. However, the stretchability of the conductor is determined by the modules of the insulating matrix. In order to eliminate the influence of shape and strain of different composite materials on the electrical conductivity, the following defined variable *G* is described to compare the conductive properties of different materials.1
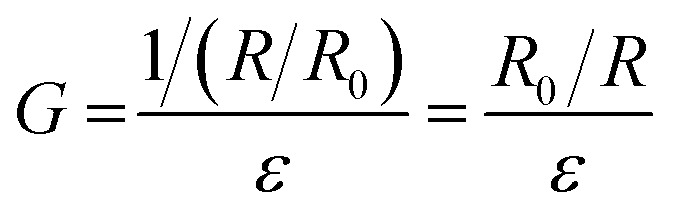
where *R*, *R*_0_ and *ε* are the resistance at the maximum strain, the initial resistance and maximum strain, respectively. The parameter *G* effectively accounts for the two factors of strain and size of different conductive materials. As shown in Fig. 6, the excellent conductivity of SELMC in this work profits from the presence of the liquid metal because liquid metal as a conductive phase is stretchable and highly conductive, which ensure a continuous conductive path during the stretching process.

**Fig. 6:**
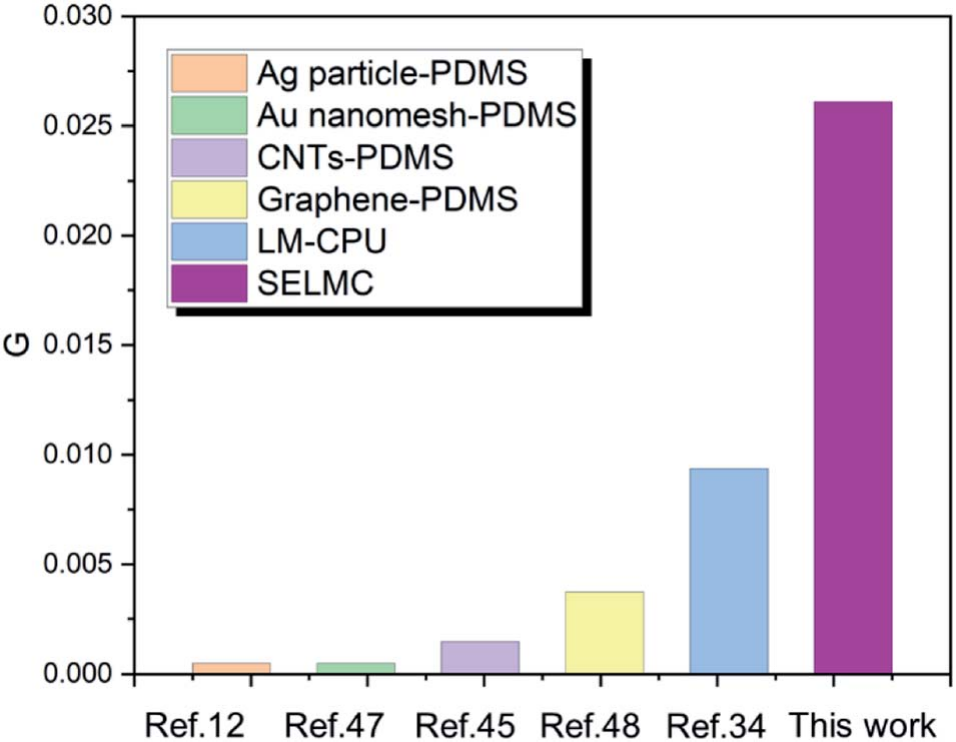
Comparison of conductance between SELMC and different conventional stretchable materials.^34,47,48^

In summary, we fabricated functional materials by using a viscous-modified PDMS and a liquid metal, which have the functions of packaging and conduction, respectively. The mechanical behaviours, thermal properties and electrical performances of the material are illustrated through bending, stretching and twisting and by applying an electric field. The results show that the relative resistance of the composite changes slightly under bending and bending–stretching cycling tests, and the increase in the relative resistance is less than 10%. During the electronical–mechanical test process, the LED could work steadily, and the liquid metal does not leak out under the twisting–bending–stretching test. The anisotropy of temperature between two faces is shown in the thermal test process. These results show that the as-prepared SELMC has excellent flexibility and stretchability. This discovery will contribute to the development of future flexible electronics. Its packaging and the anisotropy of temperature can have impacts on the field of thermal cooling technology.

## Conflicts of interest

There are no conflicts to declare.

## Supplementary Material

RA-009-C9RA06098G-s001
