# Supplementary material for: Self-encapsulation liquid metal materials for flexible and stretchable electrical conductors
Source: RSC Adv. 2019 Oct 30;9(60):35102–8. doi: 10.1039/c9ra06098g (PMC9074125; doi:10.1039/c9ra06098g)
Supplement: RA-009-C9RA06098G-s001 [file RA-009-C9RA06098G-s001.pdf]

Electronic Supplementary Material (ESI) for RSC Advances.
This journal is © The Royal Society of Chemistry 2019

Supporting information

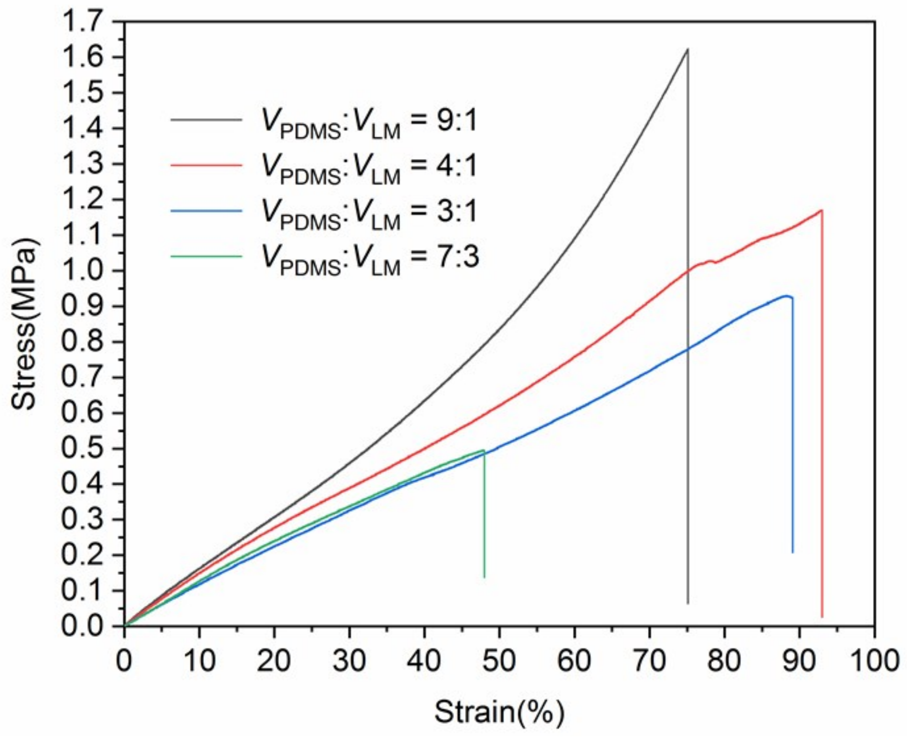

Fig. S1 The mechanical testing of different volume PDMS and liquid metal.

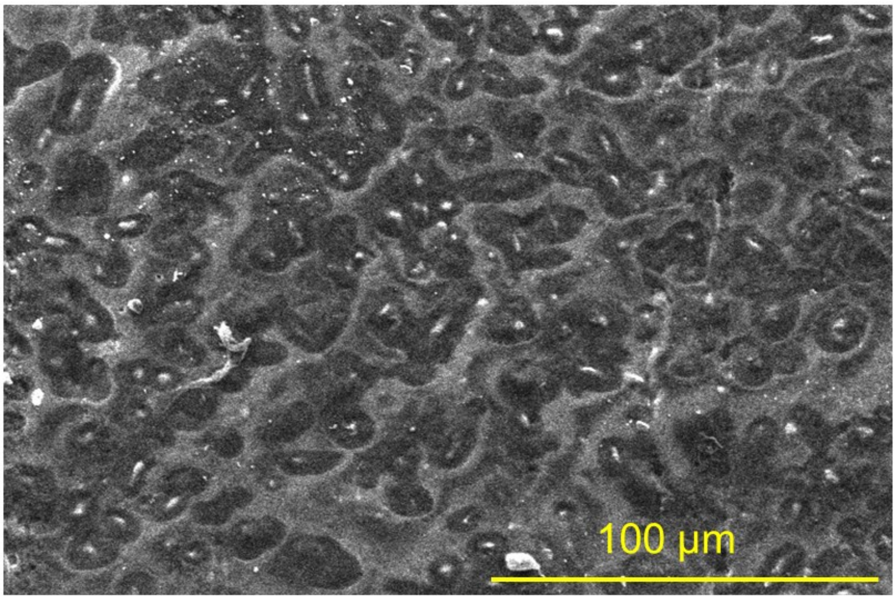

Fig. S2 The SEM photograph of SELMC for PDMS-surface.

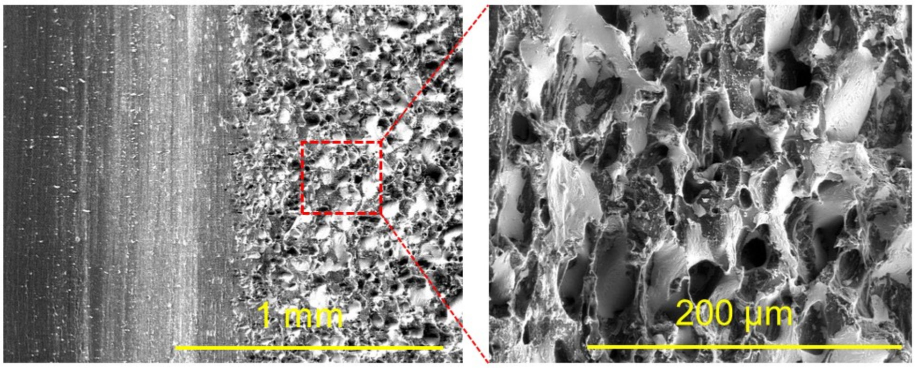

Fig. S3 The SEM photograph of PDMS @LM composite without hexane

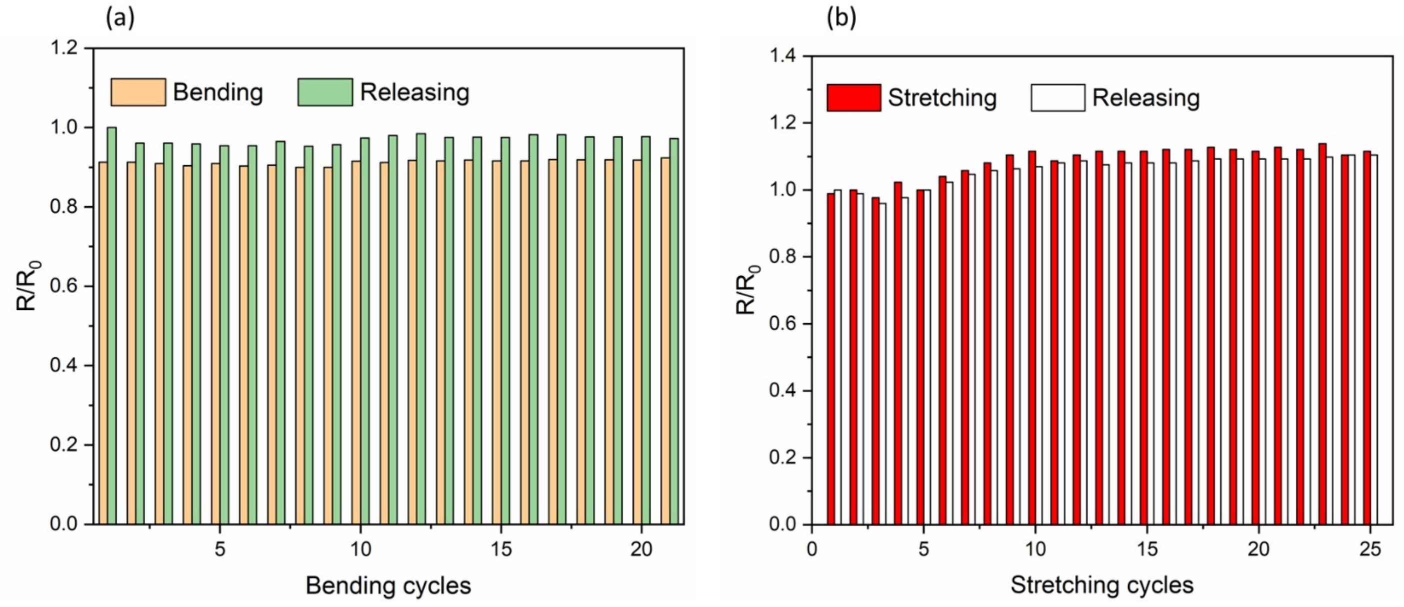

Fig. S4 The relative resistance change curves of Sample T2 at the width of 13 mm under (a) bending cycles and (b) the cooperation of bending and stretching cycles test.

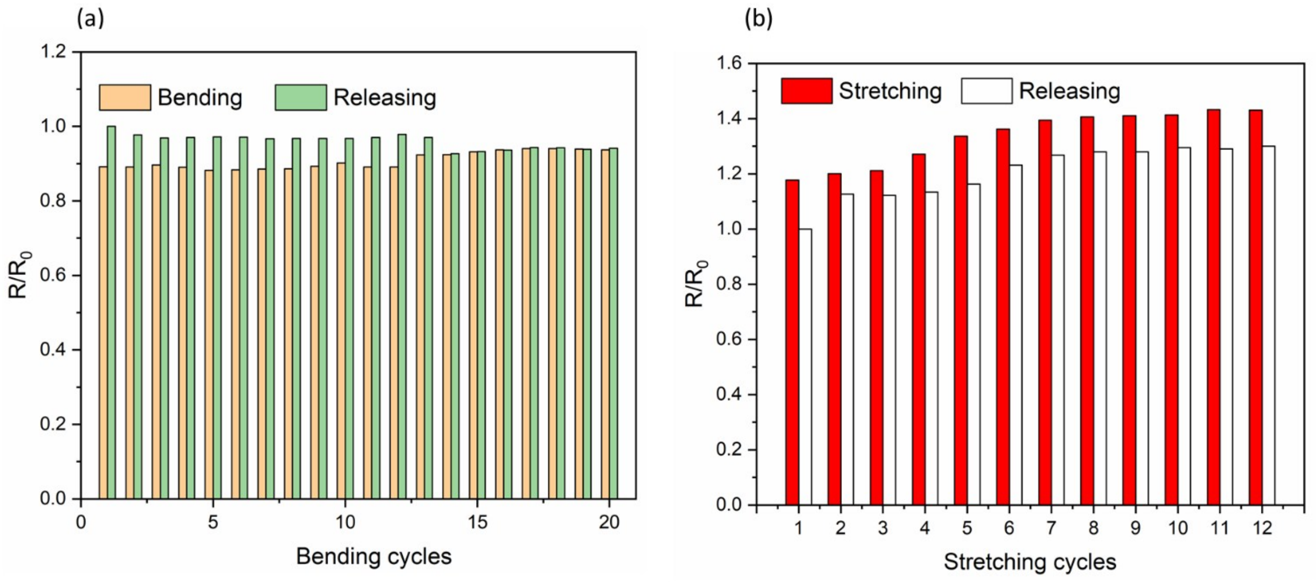

Fig. S5 The relative resistance change curves of Sample T3 at the width of 13 mm under (a) bending cycles and (b) the cooperation of bending and stretching cycles test.

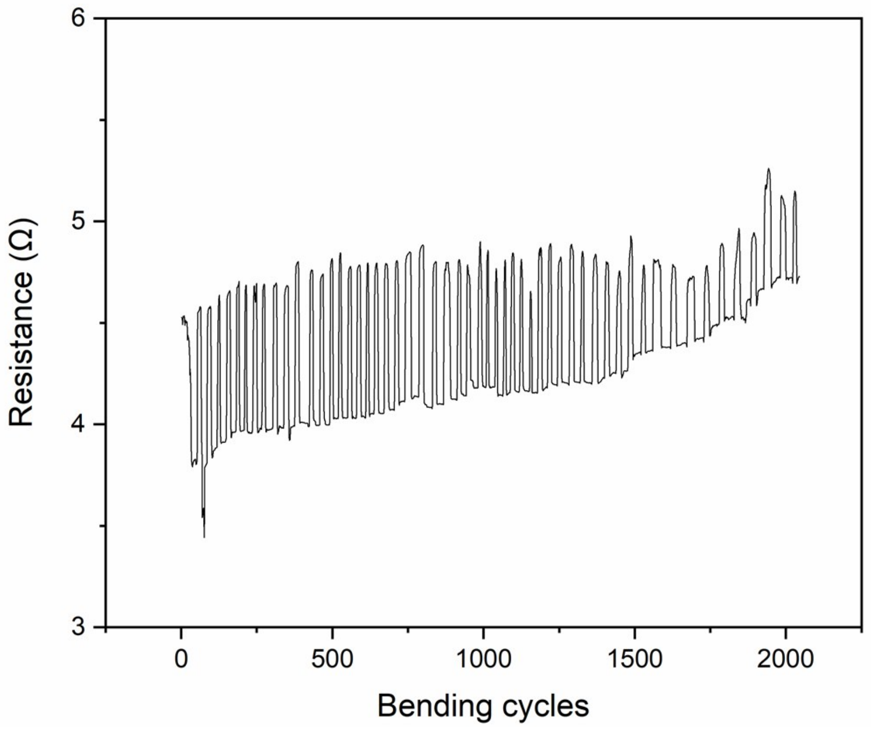

Fig. S6 Under 2000 bending cycles, the resistance value of the sample T2 at the width of 13mm.

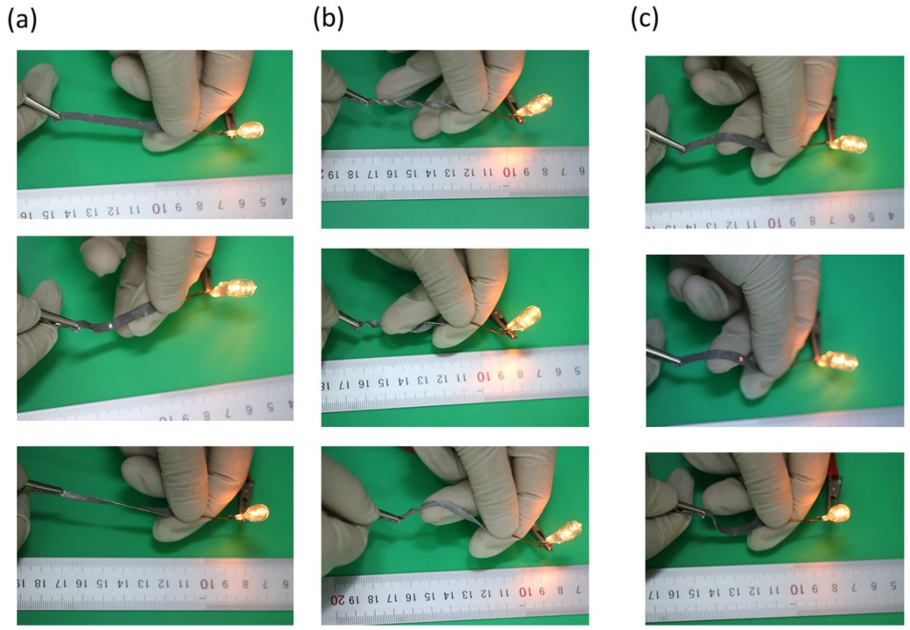

Fig. S7 The stretchable and twisting display as conductor. (a) The tensile state, (b) the twisting state, and (c) the bending state.
